# Multi-dimension metabolic prognostic model for gastric cancer

**DOI:** 10.3389/fendo.2023.1228136

**Published:** 2023-12-08

**Authors:** Wanjing Feng, Bei Xu, Xiaodong Zhu

**Affiliations:** ^1^Department of Gastrointestinal Medical Oncology, Fudan University Shanghai Cancer Center, Shanghai, China; ^2^Department of Oncology, Shanghai Medical College, Fudan University, Shanghai, China; ^3^Department of Medical Oncology, Zhongshan Hospital, Fudan University, Shanghai, China

**Keywords:** metabolic reprogramming, bio-similar cluster, gastric cancer, prognostic model, cell-cycle-related pathway

## Abstract

**Background:**

Metabolic reprogramming is involved in different stages of tumorigenesis. There are six widely recognized tumor-associated metabolic pathways, including cholesterol catabolism process, fatty acid metabolism, glutamine metabolic process, glycolysis, one carbon metabolic process, and pentose phosphate process. This study aimed to classify gastric cancer patients into different metabolic bio-similar clusters.

**Method:**

We analyzed six tumor-associated metabolic pathways and calculated the metabolic pathway score through RNA-seq data using single sample gene set enrichment analysis. The consensus clustering analysis was performed to classify patients into different bio-similar clusters by multi-dimensional scaling. Kaplan–Meier curves were presented between different metabolic bio-similar groups for OS analysis.

**Results:**

A training set of 370 patients from the Cancer Genome Atlas database with primary gastric cancer was chosen. Patients were classified into four metabolic bio-similar clusters, which were identified as metabolic non-specificity, metabolic-active, cholesterol-silence, and metabolic-silence clusters. Survival analysis showed that patients in metabolic-active cluster and metabolic-silence cluster have significantly poor prognosis than other patients (*p*=0.031). Patients in metabolic-active cluster and metabolic-silence cluster had significantly higher intra-tumor heterogeneity than other patients (*p*=0.032). Further analysis was performed in metabolic-active cluster and cholesterol-silence cluster. Three cell-cycle-related pathways, including G2M checkpoints, E2F targets, and MYC targets, were significantly upregulated in metabolic-active cluster than in cholesterol-silence cluster. A validation set of 192 gastric cancer patients from the Gene Expression Omnibus data portal verified that metabolic bio-similar cluster can predict prognosis in gastric cancer.

**Conclusion:**

Our study established a multi-dimension metabolic prognostic model in gastric cancer, which may be feasible for predicting clinical outcome.

## Background

Gastric cancer (GC) represents the fourth most common malignant neoplasm and the second leading cause of cancer-related death worldwide (1). Although surgery is considered as the only curative method for GC, perioperative chemotherapy was recommended due to the improvement of 5-year survival rate (2). However, chemotherapy resistance occurs sooner or later in these patients, with drug non-response and disease progression. Tumor-associated metabolic reprogramming is involved in different stages of tumorigenesis (3, 4). Tumor survival relies on metabolic reprogramming (5). Chemotherapy has a comprehensive relationship with tumor cell metabolism (6). Metabolic reprogramming is the result of mutations in oncogenes and tumor suppressor gene, leading to alterations of cell signaling pathway, transcriptional pathways, and posttranslational modifications (7). The conventional metabolic pathways involved in tumor-associated metabolic reprogramming included cholesterol catabolism process, fatty acid metabolism, glutamine metabolic process, glycolysis, one carbon metabolic process, and pentose phosphate process (3).

The tumor-associated metabolic reprogramming in gastric cancer is similar to other tumor species. Patients with gastric cancer have poor nutrition status and high incidence of cachexia than other tumor species. The reduced intake of nutrient intake might be an essential factor for metabolic reprogramming. Until now, a number of studies have investigated the metabolic reprogramming features in gastric cancer. First, the concentration of glucose is considered relatively depleted in gastric cancer cells than in healthy or non-malignant stomach cells (8, 9). Moreover, the high lactic acid level in gastric cancer cells is the result of sufficient oxygen and the activation of glycolysis. Second, the fatty acid level is relatively high in gastric cancer cells than in benign cells, resulting from the high activation of adipocyte lipolysis (10). The high-level fatty acid metabolism is considered as a contribution to cancer cachexia (9). Third, glutamine level is greatly depleted in gastric cancer cells, indicating the high activation of glutamine metabolism (11). The cumulative evidence showed that glutamine metabolism contributed to tricarboxylic acid cycle and nucleotide metabolism to power the tumor cells (11). In addition, other metabolism processes are also involved in gastric cancer (12).

Although the mechanism of each metabolic process in tumors has been deeply investigated, it is still not possible to generalize a metabolic pathway as pro- or anti-tumor. Of course, the role of each metabolic process is related to different tumor species. However, we hold a view that it is one-sided to focus on the influence of a simple metabolic process in tumor-associated metabolic reprogramming, due to the comprehensive associations between different metabolic processes. Therefore, this study tries to classify gastric cancer patients according to the six conventional metabolic processes in tumor-associated metabolic reprogramming and explore the difference in overall survival, molecular mechanism, and tumor microenvironment between the several metabolic subtypes.

## Methods

### Patients and specimens

RNA-sequencing and matched clinicopathological data (including histological grade, sex, stage, age, and survival data) were retrieved from the Cancer Genome Atlas (TCGA) database, the Cancer Genomics Browser of University of California Santa Cruz (https://genomecancer.ucsc.edu/).

### Metabolism score and consensus clustering analysis

The feature gene panels of cholesterol catabolism process, fatty acid metabolism, glutamine metabolic process, glycolysis, one carbon metabolic process, and pentose phosphate process were obtained from the molecular signature database (MsigDB). The enrichment scores of the six metabolic processes of each patient were analyzed by single sample gene set enrichment analysis (ssGSEA). ssGSEA estimates the relative enrichment of the six metabolic process gene sets in each sample by comparing the gene expression data of each sample with a specific gene set, which was downloaded from MsigDB.

The clustering method that we utilized in this research is called consensus clustering analysis. It is an unsupervised clustering, which use resampling method to extract data. For each sampling, the number of clusters was specified. Then, the process calculates the rationality of different cluster numbers using PAC method and identify and produce the most reasonable clustering result. In our research, the consensus clustering analysis was performed to classify patients into several bio-similar groups by multi-dimensional scaling, which classify samples into several subtypes according to six metabolic processes enrichment scores, so as to detect new disease subtypes with similar metabolic features.

### Mutant allele tumor heterogeneity

Mutant allele tumor heterogeneity (MATH) algorithm is a reliable and applicable method to measure intra-tumor heterogeneity, which has been validated and reported in gastric cancer (13). The calculation method of MATH was identified at the Broad Institute of MIT and Harvard. The calculation method includes three steps. First, each difference value of the mutant-allele fraction (MAF) was obtained from the median difference value. Second, the median absolute deviation (MAD) in R was calculated as values scaled by a factor (1.4826) to make the expected MAD of a sample from a normal distribution equals the standard deviation. Third, MATH was figured up as MATH = 100 * MAD/median. Maftools package in R was utilized to calculate MATH. Maftools package includes clustering algorithm to improve the accuracy of genomic pattern.

### Pathway enrichment analysis

Gene set enrichment analysis (GSEA) was used to analyze signaling pathway enrichment among different metabolic bio-similar groups (14). The GSEA analysis was performed according to the following standards: 1) the nominal p-value is lower than 0.05; 2) the value of NES is 1 or more than 1; 3) false discovery rate (FDR) q-value is lower than 0.25. Pathways that meet the above three criteria were considered statistically significant. The gene sets of signaling pathways were downloaded from MSigDB database (14).

### Immune pathway score

The 28 immune pathway activations of patients were analyzed by single-sample gene set enrichment analysis (ssGSEA). The activation of the immune pathways were identified by a feature panel of genes overexpressed in each kind of immune cell (15, 16). The 28 immune pathway scores include activated CD4 T cell, activated CD8 T cell, central memory CD4 T cell, central memory CD8 T cell, effector memory CD4 T cell, effector memory CD8 T cell, type 1 T-helper cell, activated dendritic cell, natural killer cell, regulatory T cell, type 2 T helper cell, macrophage, myeloid-derived suppressor cells (MDSC), activated B cell, gamma delta cell, immature B cell, T follicular helper cell, eosinophil, mast cell, and monocyte. The differences in immune pathway activation among the metabolic bio-similar groups were analyzed.

### External dataset validation

RNA-seq and clinical data were retrieved from GEO database. GSE15459 dataset was downloaded from the GEO (https://www.ncbi.nlm.nih.gov/gds/) on the GPL570 platform. The classification of patients by metabolic bio-similar features was also performed.

### Statistical analysis

Statistical methods were all analyzed using R version 3.5.1 (http://cran.r-project.org) and Stata statistical software, version 12.0 (StataCorp, College Station, TX). Patient baseline characteristics were compared among the different metabolic bio-similar groups *via* Wilcoxon rank-sum test, while multiple comparisons were performed using Kruskal–Wallis test. The association between patient characteristics and metabolic clusters was analyzed using the Spearman test. Kaplan–Meier curves were presented between different metabolic bio-similar groups for OS analysis. Log-rank test was utilized to compare the overall survival between different groups. Multivariate analysis of prognostic predictors was carried out using a Cox proportional hazards model. The differences in immune pathway activation among the metabolic bio-similar groups were analyzed using Kruskal–Wallis test. The difference in MATH among the metabolic bio-similar groups were analyzed using Wilcoxon rank sum test. A two-sided p-value <0.05 was considered significant.

## Results

### Patient characteristics and metabolic bio-similar clustering

**Figure 1:**
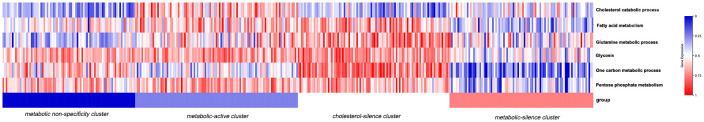
The cluster analysis according to patients’ metabolic characteristics in the training set.

A total of 370 GC patients with complete information from TCGA database were enrolled in the study as training cohort. The clinical and pathological characteristics are illustrated in **Table 1**. The enrichment scores of the six metabolic processes of each sample were analyzed by single sample gene set enrichment analysis (ssGSEA). As we have calculated the enrichment score of the six metabolic processes for each sample, then the consensus clustering analysis, an unsupervised analysis, was performed to classify patients into several bio-similar clusters according to six metabolic processes enrichment scores, so as to detect new clusters with similar metabolic features. In our study, four clusters were identified. **Figure 1** illustrated the six metabolic process enrichment of each patient and the four clustering of the patients according to metabolic process enrichment. Patients in different clusters have prominent characteristics. In cluster 3, except for cholesterol catabolism, all metabolism processes were activated. In cluster 2, all metabolism processes were activated, and cholesterol catabolism was activated only in cluster 2. In cluster 4, all metabolism processes were in low expression levels. In cluster 1, none of the metabolic processes are specifically activated or silenced. Then, we named the four clusters according to metabolic characteristics. Cluster 1 was named as metabolic non-specificity cluster. Cluster 2 was named as metabolic-active cluster. Cluster 3 was named as cholesterol-silence cluster. Cluster 4 was named as metabolic-silence cluster.

**Table 1:** Association between baseline clinicopathologic characteristics and metabolic bio-similar subtypes.

characteristics	Total	metabolic non-specificity	metabolic-active	cholesterol-silence	metabolic-silence	P value
Age						0.2944
≥65	197	49	52	53	43	
<65	173	35	50	41	47	
Sex						0.4582
Male	244	59	68	57	60	
Female	126	25	34	37	30	
Stage						0.2218
I	48	13	11	15	9	
II	118	25	42	27	24	
III	163	38	35	40	50	
IV	29	5	10	10	4	
Unknown	12	3	4	2	3	
Grade						0.0515
G1	9	1	3	2	3	
G2	132	32	42	41	17	
G3	220	49	55	50	66	
Unknown	9	2	2	1	4	

There were no significant relationships between metabolic subtypes and patient characteristics, including sex, age, stage, and histological grade (**Table 1**).

### Clinical outcome and metabolic bio-similar clustering

**Figure 2:**
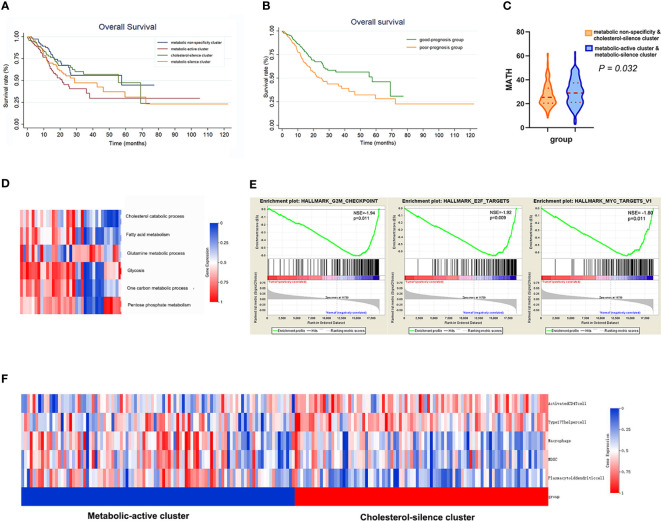
The metabolic bio-similar clusters in the training set. **(A)** Kaplan–Meier plots of overall survival in gastric cancer according to different metabolic bio-similar clusters. **(B)** Kaplan–Meier plots of overall survival in gastric cancer according to good- and poor-prognosis groups. **(C)** Difference in intra-tumor heterogeneity between different metabolic bio-similar clusters. **(D)** Metabolic characteristics in normal gastric tissues. **(E)** Gene set enrichment analysis between metabolic-active cluster and cholesterol-silence cluster. **(F)** The characteristics and heat map of the immune cell pathway activation between metabolic-active cluster and cholesterol-silence cluster.

Survival curves were estimated by Kaplan–Meier method and analyzed by log-rank test to assess the survival difference between the four metabolic subtypes. In the total of 370 patients, patients in metabolic non-specificity cluster and cholesterol-silence cluster had significantly longer OS than patients in metabolic-active cluster and metabolic-silence cluster (*p* = 0.031, **Figure 2A**). The median OS in the four metabolic subtypes were 57.4, 21.0, 55.4, and 28.7 months, respectively. We identified metabolic non-specificity cluster and cholesterol-silence cluster as good-prognosis group, while metabolic-active cluster and metabolic-silence cluster were identified as poor-prognosis group. Patients in good-prognosis group had significantly longer OS than patients in good-prognosis group (*p* = 0.007, **Figure 2B**). The multivariate Cox proportional hazards model showed that metabolic bio-similar clustering, which was categorized as good- and poor-prognosis groups, was an independent prognostic factor for OS (hazard ratio, 0.622; 95% CI, 0.441–0.879; *p* = 0.007, **Table 2**), after adjusting for clinicopathological characteristics, including age, sex, pathological stage, and histological grade.

**Table 2:** Multivariate Cox regression analysis of prognostic factors for overall survival in the training cohort.

Characteristics	Overall survival	
	Hazard ratio (95% CI)	P value
Age < 65	0.530 (0.374 - 0.751)	0.000
Female gender	0.758 (0.528 - 1.921)	0.133
Stage I	1.134 (0.467 - 2.751)	0.781
Stage II	0.199 (0.093 - 0.429)	0.000
Stage III	0.297 (0.093 - 0.429)	0.000
Stage IV	0.199 (0.164 - 0.539)	0.000
Grade 1	1.444 (0.519 - 4.015)	0.482
Grade 2	0.489 (0.119 - 2.002)	0.319
Grade 3	0.612 (0.417 - 0.900)	0.013
Metabolic bio-similar group	0.622 (0.441 - 0.879)	0.007

### Metabolic bio-similar clustering and intra-tumor heterogeneity

Intra-tumor heterogeneity was measured using mutant allele tumor heterogeneity (MATH) algorithm. MATH score is significantly associated with overall survival in gastric cancer patients, of which the result has been published. The Wilcoxon rank sum test showed that patients in metabolic-active cluster and metabolic-silence cluster had significantly higher MATH score than those in metabolic non-specificity cluster and cholesterol-silence cluster (**Figure 2C**, *p* = 0.032).

### Metabolic characteristics in normal stomach tissue

We think it is necessary to analyzed the metabolic feature of normal gastric tissues using the same method, in order to verify that tumor do affect the metabolism of gastric cells. Then, we analyzed 35 normal gastric tissue from TCGA database (**Figure 2D**). None of the metabolic processes were significantly activated or inhibited in normal gastric tissue. There was no specific metabolic characteristics in normal gastric tissues.

### Gene set enrichment analysis

In metabolic-active cluster and cholesterol-silence cluster, the patients’ outcome was different, while the metabolic characteristic was in fact similar, except for cholesterol catabolism. Patients in cholesterol-silence cluster had significantly longer OS, which was of most concern in further analysis. The difference in signaling pathways between different clusters was analyzed using GSEA. Three cell-cycle-related pathways are significantly downregulated in cholesterol-silence cluster than metabolic-active cluster, which were G2M checkpoints, E2F targets, and MYC targets (**Figure 2E**).

### Metabolic bio-similar clustering and immune pathway activation

The 28 immune pathway activations of patients were analyzed by ssGSEA. Patients in cholesterol-silence cluster had significantly longer OS than patients in metabolic-active cluster and metabolic-silence cluster. The Wilcoxon rank sum test showed that several anti-tumor immune pathway activation was significantly higher in cholesterol-silence cluster than in metabolic-active cluster, including activated CD4 T cell, type17 T-helper cell, and several pro-tumor immune pathway activations were significantly higher in metabolic-active cluster than in cholesterol-silence cluster, including macrophage, MDSC, and plasmacytoid dendritic cell (**Figure 2F**).

### External cohort validation

**Figure 3:**
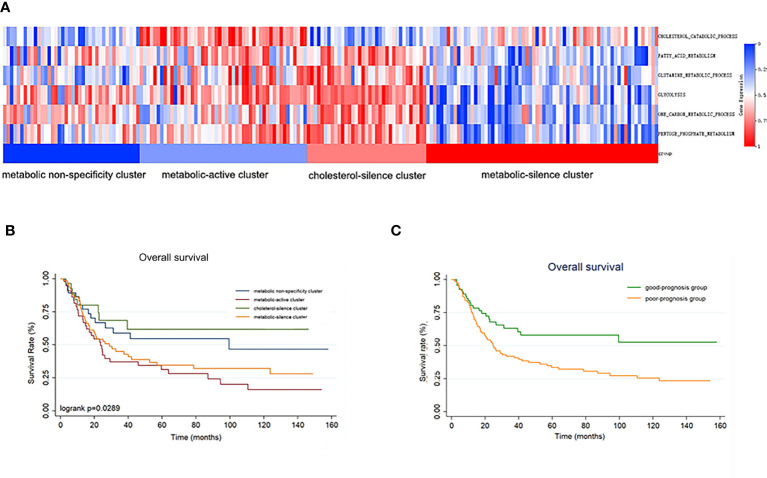
The metabolic bio-similar clusters in the validation set. **(A)** The characteristics and heat map of the metabolic pathways. **(B)** Kaplan–Meier plots of overall survival in gastric cancer according to different metabolic bio-similar clusters. **(C)** Kaplan–Meier plots of overall survival in gastric cancer according to good- and poor-prognosis groups.

We enrolled 192 gastric cancer patients with complete RNA-seq and clinical information from GSE15459 dataset to establish validation cohort. The 192 patients were classified into four clusters according to metabolic bio-similar clustering in training cohort. The enrichment of the six metabolic processes of the patients and the four clusters are illustrated in **Figure 3A**. It is obvious that the metabolic features of the four clusters were consistent with that in the training cohort.

Survival curves were also estimated by Kaplan–Meier method and analyzed by log-rank test between the four metabolic subtypes. In a total of 192 patients, patients in metabolic non-specificity cluster and cluster 3 had significantly longer OS than patients in cluster 2 and cluster 4 (*p* = 0.030, **Figure 3B**). The median OS in the four metabolic subtypes were 99.4 months, 23.6 months, not reached, and 31.2 months, respectively. We identified metabolic non-specificity cluster and cholesterol-silence cluster as good-prognosis group, while metabolic-active cluster and metabolic-silence cluster were identified as poor-prognosis group. Patients in good-prognosis group had significantly longer OS than patients in good-prognosis group (*p* = 0.007, **Figure 3C**). The multivariate Cox proportional hazards model showed that metabolic bio-similar clustering, which was categorized as good- and poor-prognosis groups, was an independent prognostic factor for OS (hazard ratio, 0.468; 95% CI, 0.291–0.559; *p* = 0.002, **Table 3**), after adjusting for clinicopathological characteristics, including age, sex, and pathological stage.

**Table 3:** Multivariate Cox regression analysis of prognostic factors for overall survival in the validation set.

Characteristics	Overall survival	
	Hazard ratio (95% CI)	P value
Age≥65	0.416 (0.557 - 1.273)	0.416
Female gender	0.890 (0.565 - 1.401)	0.616
Metabolic bio-similar group	0.468 (0.291 - 0.755)	0.002
Stage I	0.042 (0.014 - 0.122)	0.000
Stage II	0.097 (0.044 - 0.213)	0.000
Stage III	0.350 (0.219 - 0.559)	0.000

## Discussion

Our analysis used the consensus clustering analysis of RNA-sequencing data to classify gastric cancer patients according to six conventional metabolic pathways involved in tumor-associated metabolic reprogramming. Finally, patients were classified into four metabolic bio-similar clusters. Survival analysis showed that patients with all metabolic pathway activation or all metabolic pathway silencing have significantly poor prognosis than the other patients. Furthermore, an external cohort validated that the metabolic bio-similar clustering can predict the prognosis of gastric cancer patients.

The aim of the research was to assess metabolic characteristics according to multiple metabolic dimensions, rather than single metabolic dimension. The growth of tumor cells requires the metabolism of nutrients in the body for energy. Therefore, more than one metabolism pathway must be involved in this process. In previous research, the role of each metabolism pathway in tumors has been thoroughly investigated. The same metabolic pathway may play distinct roles as tumor suppressor or promotion in different kinds of tumors. For example, a prospective large sample analysis revealed that serum cholesterol level is positively associated with breast cancer, colon cancer, and prostate cancer, while it is negatively associated with liver cancer and lung cancer (17). Moreover, there are complex interactions between different types of metabolic pathways in the same tumor species. Different metabolic pathways provide each other with metabolites and energy. To our knowledge, our study is the first one to explore metabolic clusters in gastric cancer and revealed out four metabolic bio-similar clusters in gastric cancer.

In metabolic-active cluster and cholesterol-silence cluster, the patients’ outcome was different, while the metabolic characteristic was in fact similar, except for cholesterol catabolism. Patients in cholesterol-silence cluster had significantly longer OS, which was of most concern for further analysis. Cholesterol is an important structure for cell surface, and rapid cell proliferation requires more cholesterol synthesis. The GSEA analysis showed that three cell-proliferation-related pathways, namely, G2M checkpoints, E2F targets, and MYC targets, were significantly downregulated in cholesterol-silence cluster. What G2M checkpoints, E2F targets, and MYC targets have in common is that they all play a pivotal role in cell cycle progression and cell division. The overexpression of oncogenic G2/M checkpoint may lead to poor prognosis (18, 19). The cyclin-dependent kinase (CDK)–retinoblastoma gene (RB)–E2F axis plays an important role in cell cycle progression, which controls genome replication and accurate cell division cycle (20). In transformed cells, altered expression of E2F can increase E2F activity and induce replicative stress and high rates of proliferation (21). E2F expression and/or E2F targets elevated expression have been related to poor prognosis in tumors (22, 23). Earliest research suggested that the function of c-Myc was to promote cell proliferation. Later, it was uncovered that MYC target genes could encode proteins that regulate cell cycle (24). Cancer cells, as fast-proliferating cells, require high level of cholesterol metabolism for membrane biogenesis (25, 26). We hypothesize that downregulation of cholesterol may influence membrane synthesis, resulting in inhibition of cell proliferation, which might be the mechanism of better prognosis of cholesterol-silence cluster. For metabolic-active cluster, it is obvious that tumor metabolism is the most active in these patients, which might be a manifestation of rapid tumor proliferation and lead to poor prognosis.

Moreover, we also found that immune suppression played an important role in survival difference between metabolic-active cluster and cholesterol-silence cluster. We compared differences in immune cell pathway activation between metabolic-active cluster and cholesterol-silence cluster. The results showed that several pro-tumor immune cells pathways were significantly activated in metabolic-active cluster and several anti-tumor immune cells pathways in cholesterol-silence cluster. Several studies have reported that high cholesterol expression in tumor cells is related to the loss of anti-tumor effects in immune cells. High level of cholesterol can protect tumor cells from immune surveillance (27). The high production of cholesterol from tumor cells can promote the expression of suppressive immune checkpoint in T cells (28).

There was no specific metabolic process activation or inhibition in in metabolic non-specificity cluster. We calculated enrichment scores of six metabolic processes in normal gastric tissue using the same method. It was obvious that non-metabolic process was significantly activated or inhibited in normal gastric tissue. The similarity of normal gastric tissue and tumor tissue metabolic non-specificity cluster might be an important reason why patients in metabolic non-specificity cluster had better prognosis. Patients in metabolic-silence cluster had significantly poor prognosis. In general, it is considered that low level metabolism provides insufficient energy for tumor proliferation and metastasis. However, our study found that patients with low level metabolism had significant poor prognosis. In pancreatic cancer, metabolic bio-similar cluster analysis also reported that patients in metabolic quiescent cluster had relatively poor prognosis (29). We suspect that these patients may not have enough nutrients to consume due to their poor nutritional status. Poor nutritional status is related to poor prognosis in gastric cancer, which might be the reason why patients in metabolic-silence cluster had poor prognosis.

After analyzing the characteristics of different subtypes one by one, let us sort them out again. First, patients in both metabolic-active and metabolic-silence clusters have relatively poor prognosis. It indicated that hyperactive metabolism promoted tumor proliferation, while excessive inhibition of metabolism might result in inadequate energy supply and poor prognosis. Second, patients in cholesterol-silence cluster showed good prognosis. According to our further analysis, it may be due to insufficient raw material cholesterol for cell membrane synthesis. Lastly, patients in metabolic non-specificity cluster also showed good prognosis, which might be due to the metabolic similarity to normal gastric tissue. We hope that the metabolic clusters provided by our study will provide insights into the design of targeted therapies in the future.

The present research had several limitations. First, we chose six of the most recognized metabolic pathways for our study. However, there must be other metabolic pathways that contribute to tumor progress, which might not have been uncovered yet or thought to be less important in tumor metabolism. Second, in metabolic non-specificity cluster, we failed to distinguish the metabolic characteristics of these people, which requires further analysis.

## Conclusion

Our study established a multi-dimension metabolic prognostic model in gastric cancer. Patients were divided into four metabolic bio-similar clusters, including metabolic non-specificity cluster, metabolic-active cluster, cholesterol-silence cluster, and metabolic-silence cluster. Patients in metabolic-active cluster and metabolic-silence cluster had poorer prognosis. To conclude, multi-dimension metabolic prognostic model is a feasible method to for predict clinical outcome in gastric cancer.

## Compliance with ethical standards

All analysis in this research based on the data from TCGA and GEO public database that met ethical guidelines.

## Data availability statement

The original contributions presented in the study are included in the article/**Supplementary Material**. Further inquiries can be directed to the corresponding authors.

## Author contributions

Conceptualization, XZ; Data curation, BX; Funding acquisition, XZ; Methodology, WF; Writing – original draft, WF; Writing – review & editing, BX and XZ. All authors contributed to the article and approved the submitted version.
