# Supplementary material for: Multi-dimension metabolic prognostic model for gastric cancer
Source: Front Endocrinol (Lausanne). 2023 Dec 8;14:1228136. doi: 10.3389/fendo.2023.1228136 (PMC10748418; doi:10.3389/fendo.2023.1228136)
Supplement: Supplementary file 1 [file DataSheet_1.docx]

Consensus Cluster

library(ConsensusClusterPlus)

setwd("F://")

data <- read.table(file = "XXX.txt", sep = "\t", header = T, stringsAsFactors = F, row.names = 1, check.names = F)

data2 <- data[apply(data, 1, function(x){sum(is.na(x)) < ncol(data)/2}),]

data2 <- as.matrix(data2)

res <- ConsensusClusterPlus(data2, maxK = 10, reps = 1000, pItem = 0.8, pFeature = 1, distance="euclidean", clusterAlg = "pam", corUse = "complete.obs", seed=123456, plot="png", writeTable=T, verbose=T)

calcICL(res,title="untitled_consensus_cluster",plot="png", writeTable=FALSE)

MATH

library(maftools)

annovarToMaf(annovar, Center = NULL, refBuild = "hg19", tsbCol = NULL, table = "refGene", basename = NULL, sep = "\t", MAFobj = FALSE, sampleAnno = NULL)

setwd("F://")

var_maf<-read.maf(maf ="XXX.maf")

getGeneSummary(var_maf)

getSampleSummary(var_maf)

getClinicalData(var_maf)

plotmafSummary(maf=var_maf, rmOutlier=TRUE, addStat = 'median')

getFields(var_maf)

var_maf@data$VAF<-var_maf@data$t_alt_count/var_maf@data$t_depth

plotVaf(maf=var_maf,vafCol = "VAF")

somaticInteractions(maf = var_maf, top = 25, pvalue = c(0.05, 0.1))

head(var_maf@data$Tumor_Sample_Barcode)

math <- math.score(maf = var_maf, vafCol = VAF)

write.table(math,"XXX.txt",sep = "\t")

ssGSEA

rm(list=ls())

setwd("test://")

library(GSEABase)

library(GSVAdata)

list <- getGmt("test.geneset")

head(names(list))

list[[names(list)[1]]]

mydata <- read.table(file = "test.txt",header=T)

name<-mydata[,-1]

data<-mydata[!apply(name,1,sum)==0,]

data$median=apply(data[,-1],1,median)

data=data[order(data$Samples,data$median,decreasing=T),]

data=data[!duplicated(data$Samples),]

rownames(data)=data$Samples

uni_matrix<-data[,grep('\\d+',colnames(data))]

uni_matrix<-log2(uni_matrix+1)

colnames(uni_matrix)<-gsub('X','',gsub('\\.','\\-',colnames(uni_matrix)))

uni_matrix<-uni_matrix[,order(colnames(uni_matrix))]

uni_matrix= as.matrix(uni_matrix)

library(Biobase)

library(genefilter)

library(limma)

library(RColorBrewer)

library(GSVA)

gsva_matrix<- gsva(uni_matrix, list, method='ssgsea',kcdf='Gaussian',abs.ranking=TRUE)

library(pheatmap)

gsva_matrix1<- t(scale(t(gsva_matrix)))

gsva_matrix1[gsva_matrix1< -2] <- -2

gsva_matrix1[gsva_matrix1>2] <- 2

normalization<-function(x){return((x-min(x))/(max(x)-min(x)))}

nor_gsva_matrix1 <- normalization(gsva_matrix1)

write.table(nor_gsva_matrix1,"test.txt",sep = "\t")
